# Epigallocatechin gallate (EGCG) partially prevents elastase-induced mechanical and microstructural changes in the mouse ascending aorta in vitro^☆^

**DOI:** 10.1016/j.jmbbm.2026.107340

**Published:** 2026-01-08

**Authors:** Luis A. Castro, Dongfang E. Chen, Aidan O’Scannlain, Krashn K. Dwivedi, Keshav A. Kailash, Jacob Rother, Christie L. Crandall, Robyn A. Roth, Carmen M. Halabi, Jessica E. Wagenseil

**Affiliations:** a Departments of Mechanical Engineering and Materials Science, United States; b Biomedical Engineering, Washington University in St. Louis, St. Louis, MO, United States; c Department of Pediatrics, Division of Nephrology, Washington University School of Medicine, St. Louis, MO, United States

**Keywords:** Elastin, Elastic fibers, Polyphenols, Aorta, Mechanics, Aneurysm

## Abstract

Elastic fibers are critical for proper mechanical function of large arteries such as the aorta. Fragmentation, degradation, or reduced amounts of elastic fibers are associated with aortic diseases such as stiffening-induced hypertension and aneurysms. Epigallocatechin gallate (EGCG) is a plant-based polyphenol that has been shown to increase elastic fiber synthesis, preventing stiffening-induced hypertension and alleviating abdominal aortic aneurysms. EGCG is similar in structure to another polyphenol, pentagalloyl glucose, that has been shown to increase elastic fiber synthesis and prevent elastic fiber degradation. The effects of EGCG on elastic fiber degradation have not been previously investigated. In this study, elastase (ELA) was used to degrade elastic fibers in the mouse ascending aorta and the preventative and restorative potential of EGCG was determined by characterizing the passive, circumferential mechanical behavior and microstructural organization of the aortic wall. EGCG treatment alone had no effect on the mechanical behavior or microstructural organization of the aortic wall. ELA treatment alone resulted in increased aortic diameter, altered aortic compliance, reduced low modulus, increased high modulus, and decreased density of the elastic fiber layers in the wall. EGCG as a preventative treatment before ELA partially prevented the changes in mechanical behavior and wall structure observed with ELA. EGCG as a restorative treatment after ELA did not prevent the changes in mechanical behavior and wall structure associated with ELA. These results suggest that EGCG may be an effective preventative pharmaceutical treatment option for cardiovascular diseases that are characterized by elastic fiber degradation.

## 1. Introduction

Elastic fibers are complex structures that contain elastin, the primary protein that gives tissues elastic properties (Baldwin et al., 2013). Elastic fibers allow large arteries, such as the aorta, to respond elastically to large, cyclic pressure and volume changes (Wagenseil et al., 2009). Decreased amounts and fragmentation or degradation of elastic fibers are associated with cardiovascular diseases such as stiffness-induced hypertension (Wagenseil et al., 2012) and aneurysms (Yanagisawa et al., 2020). Identifying pharmaceutical treatments that can prevent or restore elastic fibers to their normal amounts and mechanical integrity would significantly impact cardiovascular disease management and outcomes (Heinz, 2021).

Epigallocatechin gallate (EGCG) is a polyphenolic compound related to several other catechins that are believed to be responsible for the health benefits of green tea (Nagle et al., 2006). EGCG, catechin, and another polyphenolic compound, pentagalloyl glucose (PGG), facilitated an early self-assembly step that is necessary for elastic fiber synthesis when individually combined in vitro with tropoelastin (the soluble precursor to elastin) (Sinha et al., 2014). EGCG and PGG also increased elastic fiber synthesis in cell culture (Sinha et al., 2014; Ellis et al., 2022; Chowdhury et al., 2021) and in the arteries of treated mice (Ellis et al., 2024; Wang et al., 2021). EGCG and PGG were effective at attenuating progression of abdominal aortic aneurysms in rats, but possibly through different mechanisms. PGG bound to elastic fibers and stabilized them against inflammatory degradation (Isenburg et al., 2007), while EGCG regenerated elastic fibers and reduced matrix metalloproteinase activity that may contribute to elastic fiber degradation (Setozaki et al., 2017).

Preventing elastic fiber degradation and promoting elastic fiber deposition are both possible approaches to addressing cardiovascular diseases associated with compromised elastic fibers. However, due to the complex spatial and temporal regulation of elastic fiber assembly, neo-synthesis of elastic fibers has typically been viewed as the more challenging approach (Cocciolone et al., 2018). On the other hand, preventing degradation of elastic fibers has shown promise using PGG treatments in pulmonary hypertension (Kucherenko et al., 2023), emphysema (Parasaram et al., 2021), thoracic aortic aneurysm (Crandall et al., 2022), and abdominal aortic aneurysm (Isenburg et al., 2007). Although PGG and EGCG are both polyphenols and have similar effects on elastic fiber synthesis (Sinha et al., 2014), the role of EGCG in preventing elastic fiber degradation is unknown.

In this study, we investigated the potential of EGCG to prevent or restore elastic fiber mechanical and structural changes caused by in vitro elastase (ELA) treatment in the mouse ascending aorta. ELA treatment was optimized to produce mild elastic fiber degradation and aortic dilation, as observed in aortic aneurysm. EGCG was used alone, before elastase as a preventative treatment (EGCG + ELA), and after elastase as a restorative treatment (ELA + EGCG). Changes in the mechanical behavior of the aorta were quantified through passive, circumferential mechanical testing for the untreated (UNT) and treated (ELA, EGCG, EGCG + ELA or ELA + EGCG) states. Structural changes were characterized by multiphoton imaging and transmission electron microscopy (TEM).

## 2. Materials and methods

### 2.1. Mice and aortic treatment groups

**Fig. 1.**
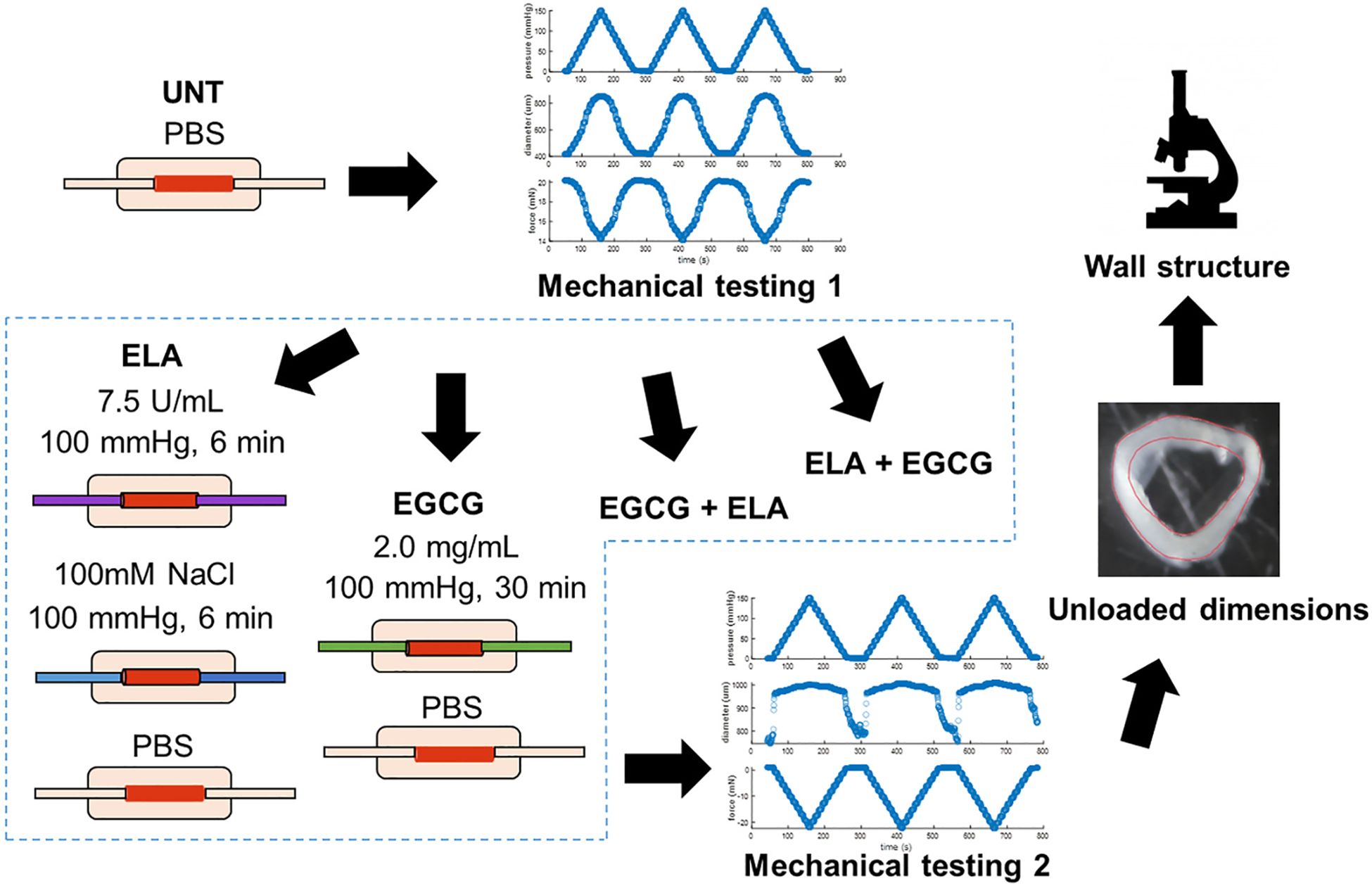
Mouse ATAs were cannulated onto a pressure myograph and mechanically tested in the untreated (UNT) condition to collect pressure, diameter, and axial force data at the approximate in vivo axial stretch ratio. The ATAs were then placed in one of four treatment groups (ELA, EGCG, EGCG + ELA, or ELA + EGCG) and mechanically tested again. After mechanical testing, cross-sectional rings were cut to measure unloaded dimensions and then processed for imaging of the wall structure. Adapted from Crandall et al. (Crandall et al., 2022).

C57BL6/J male mice (Jackson Labs, #000664) ages 13–16 weeks were euthanized by carbon dioxide inhalation and the ascending thoracic aorta (ATA) was removed (N = 25 total). Male mice were used to reduce variation in aortic size and mechanical behavior associated with sex (Hawes et al., 2020). All animal procedures were approved by the Institutional Animal Care and Use Committee. ATAs were stored at 4 °C in phosphate-buffered saline (PBS) for up to 4 days before use (Amin et al., 2011). For mechanical testing, ATAs were mounted in a pressure myograph (110P, Danish Myotechnology) in a PBS bath at 37 °C and tied onto cannulae using 7–0 sutures. The unloaded length of the ATA was determined as the length at which it was not obviously buckled and the axial force began to increase when it was stretched. Untreated (UNT) mechanical testing data (N = 20) were obtained (as described in the next section) and then the aorta was placed in one of four treatment groups: ELA, EGCG, EGCG + ELA, or ELA + EGCG (Fig. 1) (N = 5/group). Drug treatments were administered by infusing treatments into the ATA lumen and pressurizing the ATA to 100 mmHg to mimic in vivo conditions and facilitate transmural drug transport. ELA (Elastin Products Company, EC134GI) was used at 7.5 U/mL in PBS, held in the lumen for 6 min, and followed by 100 mM NaCl for 6 min to stop the enzymatic activity (Crandall et al., 2022). EGCG (Sigma, E4143) was used at 2 mg/ml in PBS and held in the lumen for 30 min. ELA and EGCG dosage and treatment times were optimized with the assumption of an effect: mild dilation for ELA and some prevention of the dilation for EGCG + ELA. After treatment, the ATA lumen was rinsed with PBS, the unloaded length was redetermined, and mechanical testing data were obtained for the treated condition. After all mechanical testing, the ATA was removed from the myograph, cut into rings to obtain unloaded dimensions, and then fixed and stored for imaging (Fig. 1). Additional UNT ATAs (N = 5) were also processed for imaging.

### 2.2. Mechanical testing

**Fig. 2.**
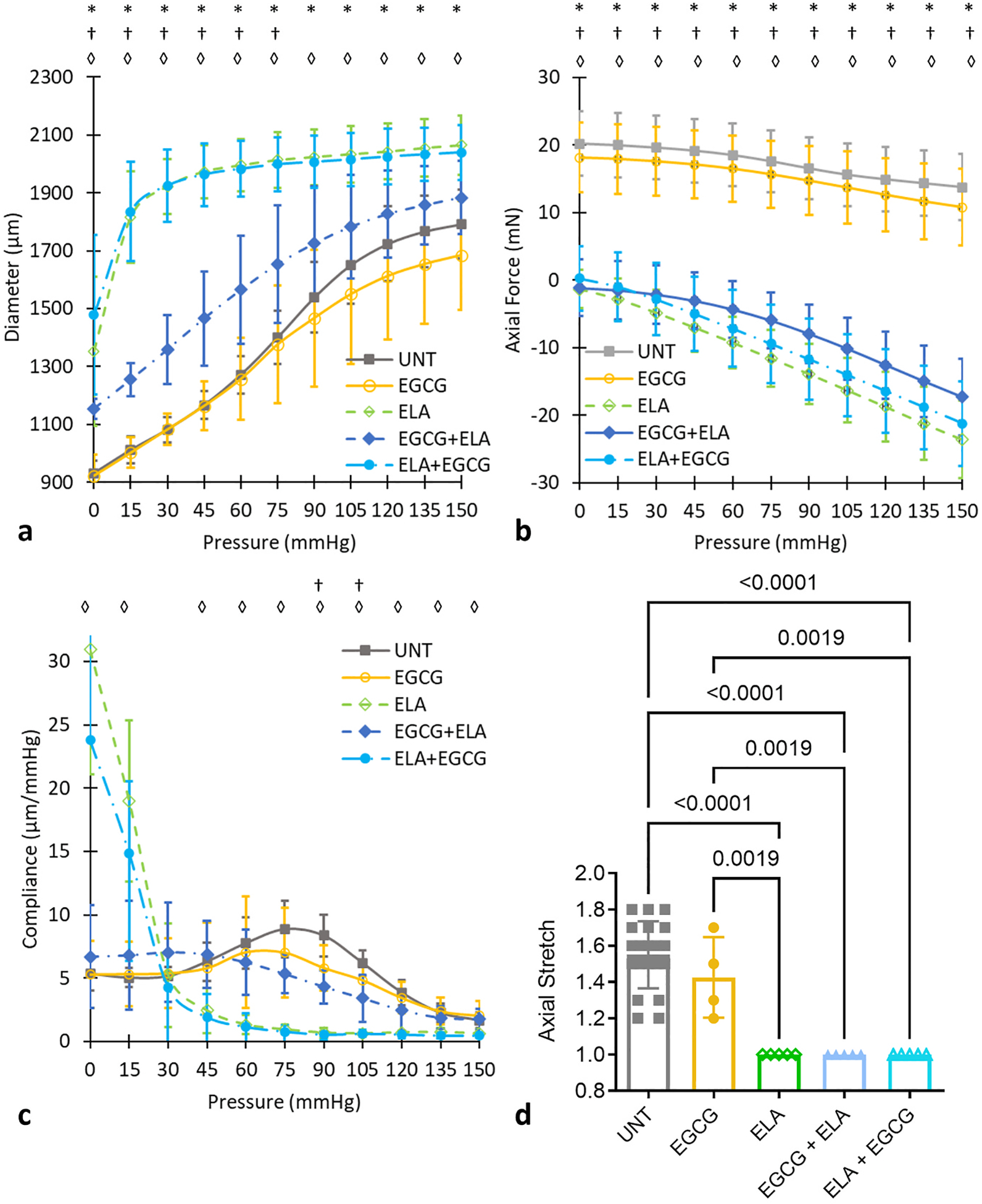
Average diameter-pressure (a), axial force-pressure (b), and compliance-pressure (c) at the approximate in vivo axial stretch ratio are shown with comparisons made to the UNT group using one-way ANOVA with Tukey’s posthoc test and p-values <0.05 noted by *, †, ◊ for ELA, EGCG + ELA, ELA + EGCG, respectively. The approximate in vivo axial stretch ratios (d) are shown for each group with comparisons between all groups by one-way ANOVA with Tukey’s posthoc test and significant p-values noted. No significant differences were found between EGCG and UNT ATAs for any of the data shown. Mean ± SD. N = 20 for UNT, N = 5/group for all others.

Mechanical testing protocols were carried out as described previously (Dwivedi et al., 2024). Briefly, ATAs were preconditioned and then inflated three times between 0 and 150 mmHg while held at the approximate in vivo stretch ratio. The in vivo stretch ratio was chosen as the stretched length at which the axial force was approximately constant when the ATA was inflated from 0 to 150 mmHg (Ferruzzi et al., 2013). For ELA, EGCG + ELA, and ELA + EGCG groups the ATA tore when stretched to a length where the axial force was constant during inflation, so all ATAs in these groups were all tested at an axial stretch ratio of 1.0. The lumen pressure (*P*), loaded outer diameter (*d*_*o*_), and axial force (*f*) were recorded throughout the testing protocol. Each ATA underwent UNT mechanical testing, was treated with one of the four treatment group options, and then was tested again. After testing, the ATAs were removed from the myograph and 200–300 μm thick rings were cut and imaged. Outer and inner circumferences of three rings were traced and ellipses were fit to determine the average unloaded outer (*D*_*o*_) and inner (*D*_*i*_) diameters using Image J (NIH). One ATA in the EGCG group ripped before rings were cut, therefore N = 4 for EGCG unloaded dimensions. For stretch and stress calculations, the average unloaded outer and inner diameters for the EGCG group were used as the unloaded diameters for the one EGCG ATA that did not have rings. UNT rings were not available due to the experimental design. As UNT and EGCG pressure-diameter curves were not significantly different from each other (Fig. 2A), average unloaded dimensions from the EGCG group were used as the UNT unloaded dimensions.

### 2.3. Mechanical data analysis

The third inflation cycle was used for analysis, if possible. Compliance was calculated from the average change in diameter per 15 mmHg pressure step. The normal stretch ratios (*λ*) in each direction (*θ* = *circumferential, z* = *axial,r* = *radial*) are,

(1) $$\lambda_{\theta \theta }=\frac{1}{2}\left(\frac{d_{i}}{D_{i}}+\frac{d_{o}}{D_{o}}\right),\lambda_{zz}=\frac{l}{L},\lambda_{rr}=\frac{1}{\lambda_{\theta \theta }\lambda_{zs}},$$

where *d*_*i*_ is the deformed inner diameter and was calculated assuming incompressibility, *l* is the loaded length, and *L* is the unloaded length. The mean stresses (*σ*) in the circumferential and axial directions were determined assuming inflation and stretch of a cylindrical aorta with no shear (Humphrey, 2002),

(2) $$\sigma_{\theta \theta }=\frac{Pd_{i}}{d_{o}-d_{i}},\sigma_{zz}=\frac{4f+Pd_{i}^{2}}{\pi \left(d_{o}^{2}-d_{i}^{2}\right)}.$$

### 2.4. Circumferential material stiffness

The circumferential stress-stretch curve for the ATA shows a nonlinear response that can be described by piecewise linear functions (Kim et al., 2017). The slopes of the lines in the low and high stretch regions are attributed to the circumferential material stiffness of the ATA provided by the elastic and collagen fibers, respectively (Dwivedi et al., 2024). Linear fits in the low (0–15 mmHg) and high (120–150 mmHg) stretch regions were performed using custom MATLAB scripts (Dwivedi et al., 2023). The ranges were chosen as linear regions at the beginning and end of the pressure-diameter curves for all groups (Fig. 2a). The resulting slopes are reported as low and high moduli.

### 2.5. Imaging

For multiphoton imaging, ATA rings were fixed in 4 % paraformaldehyde, flash frozen in O.C.T. Compound, and cut into 10 μm cross-sections using a cryotome. Sections were placed on bonded slides, cover slipped, and imaged on a Leica Sp-8 DIVE Multiphoton microscope. Z-stacks were captured for elastin autofluorescence (excitation: 880 nm, emission: 495–540 nm) and collagen second harmonic generation (excitation: 880 nm, emission: 420–460 nm) (Crandall et al., 2022).

TEM of ATA rings was accomplished following a previously reported protocol with minor modifications (Davis et al., 2017). Briefly, ATA rings were fixed in 10 % formalin for at least 30 days and further fixed in 2.5 % glutaraldehyde for approximately 30 days. Samples were then post-fixed with 1.25 % osmium tetroxide and sequentially stained with 2 % tannic acid and 6 % uranyl acetate with extensive rinsing between each stain. ATAs were dehydrated through a graded ethanol series followed by rinses of propylene oxide before infiltration with a graded series of propylene oxide/araldite resin and then left to infiltrate overnight in 100 % araldite resin. Samples were then transferred into fresh resin and embedded in silicon molds followed by polymerization at 60 °C. Sample blocks were sectioned with an ultramicrotome at 60 nm thickness. Sections were collected on formvar coated slot grids and post stained with 6 % uranyl acetate and lead citrate. Sections were visualized on a JEOL 1400 electron microscope and imaged with an AMT XR111 digital camera.

### 2.6. Statistical analysis

Statistical analysis was performed in Prism (GraphPad). Mean ± standard deviation (SD) values are reported. Experimental groups were compared using one-way ANOVA with Tukey’s multiple comparisons for the mechanical testing data and calculated values. P < 0.05 was considered significant. Imaging data was compared qualitatively.

## 3. Results

### 3.1. Passive, circumferential mechanical behavior

Passive, circumferential mechanical behavior for each treatment group was investigated by subjecting each ATA to inflation cycles at the approximate in vivo axial stretch. Average pressure-diameter, pressure-axial force, and pressure-compliance behavior are shown in Fig. 2a–c. The approximate in vivo axial stretch ratio for each group is shown in Fig. 2d. The approximate in vivo axial stretch ratio was chosen so that the axial force was approximately constant with increasing pressure for UNT and EGCG ATAs (Fig. 2b). The ELA treated ATAs could not be tested at the same axial stretch ratios as UNT and EGCG ATAs without tearing, so the axial stretch ratio for those groups was always 1.0. There are no significant differences between UNT and EGCG ATAs for the pressure-diameter (Fig. 2a), pressure-axial force (Fig. 2b), or pressure-compliance (Fig. 2c) behavior, demonstrating that EGCG alone does not alter mechanical behavior of the ATA.

There are significant increases in the ATA diameter at each applied pressure for ELA and ELA + EGCG compared to UNT with 15–79 % increases for ELA and 14–81 % increases for ELA + EGCG (Fig. 2a). EGCG + ELA ATA diameter is only significantly different compared to UNT ATA in the pressure range from 0 to 75 mmHg with increases of 5–26 % (Fig. 2a). The axial force was significantly decreased at every pressure for ELA, EGCG + ELA, and ELA + EGCG ATAs compared to UNT ATA with decreases between 100 and 271 % (Fig. 2b). However, this is mostly due to the reduced axial stretch in all ELA treated ATAs (Fig. 2d). Compliance for ELA and EGCG + ELA ATAs increased 277–478 % compared to UNT ATA at low pressures and decreased 61–92 % at high pressures (Fig. 2c). Compliance for EGCG + ELA ATA was only significantly different from UNT ATA at 90 and 105 mmHg with decreases of 45–48 %. Together, these results indicate that 1) ELA treatment affects the circumferential and axial mechanical behavior of the ATA; 2) these changes are not restored by ELA + EGCG treatment; and 3) these changes are partially prevented by EGCG + ELA treatment.

### 3.2. Unloaded dimensions

**Fig. 3.**
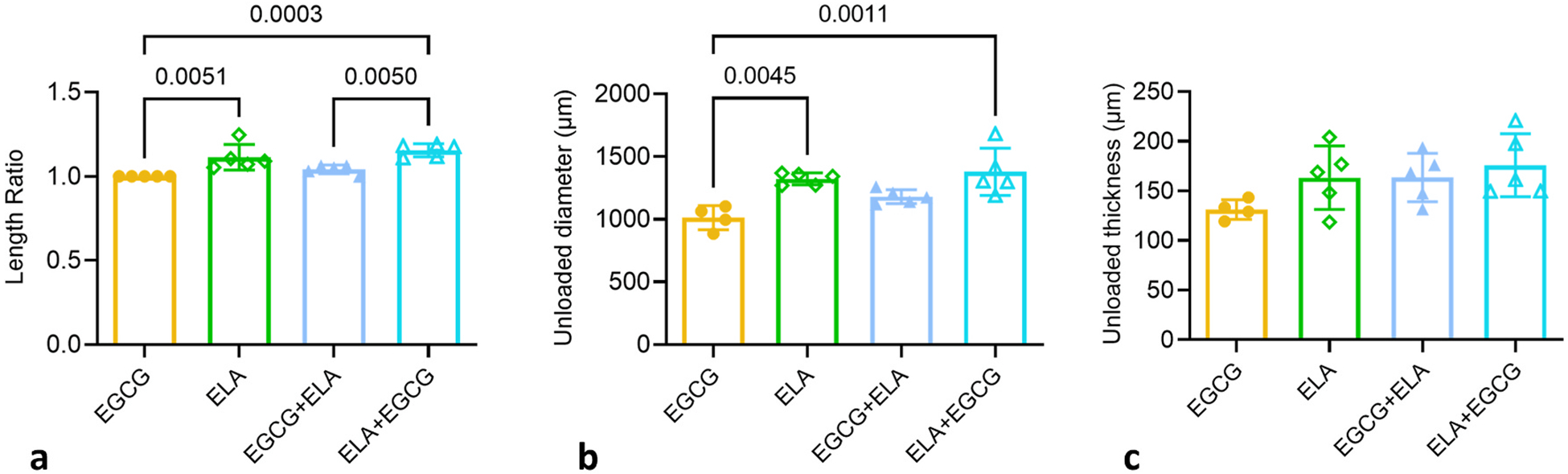
Average unloaded length ratios (after/before treatment) (a), unloaded outer diameters (b), and unloaded thicknesses (c) of the ATAs for the four treatment groups. Because unloaded outer diameters and thicknesses were measured from cut rings at the conclusion of all mechanical testing, these dimensions are not available for UNT ATAs. Comparisons between groups from one-way ANOVA with Tukey’s posthoc test and significant p-values are shown. Mean ± SD. N = 4–5/group.

The ratio of the unloaded length after treatment to the unloaded length before treatment was calculated for each ATA. The length ratio did not change after EGCG or EGCG + ELA treatments (near 1.0) (Fig. 3a). The length ratio was increased after ELA and ELA + EGCG treatments (>1.0) (Fig. 3a). Compared to EGCG ATA (which had similar behavior to UNT ATA in Fig. 2), the length ratios of ELA and ELA + EGCG are significantly increased, while the length ratio of EGCG + ELA is similar. The unloaded outer diameters and thicknesses were measured for the ATAs after all mechanical testing; hence these dimensions are only available for the treated groups. Compared to EGCG ATA, the unloaded outer diameters of ELA and ELA + EGCG ATAs are significantly increased by 31 % and 36 %, while the unloaded outer diameter of EGCG + ELA is similar (Fig. 3b). These results suggest that EGCG + ELA prevents ATA lengthening and diameter dilation associated with ELA treatment, but ELA + EGCG does not. The unloaded thicknesses did not change with drug treatment (Fig. 3c). Due to the experimental design, we do not have unloaded outer diameters and thicknesses for UNT ATAs. Since the UNT and EGCG pressure-diameter curves were similar (Fig. 2a), the average unloaded outer diameters and thicknesses for EGCG ATAs were used as the unloaded dimensions for all UNT ATAs for stretch and stress calculations. For the drug treatment groups, the unloaded dimensions for each individual ATA were used for stretch and stress calculations, except in the case of one EGCG ATA where the average for the group was used because individual measurements were not available.

### 3.3. Stress-stretch behavior

**Fig. 4.**
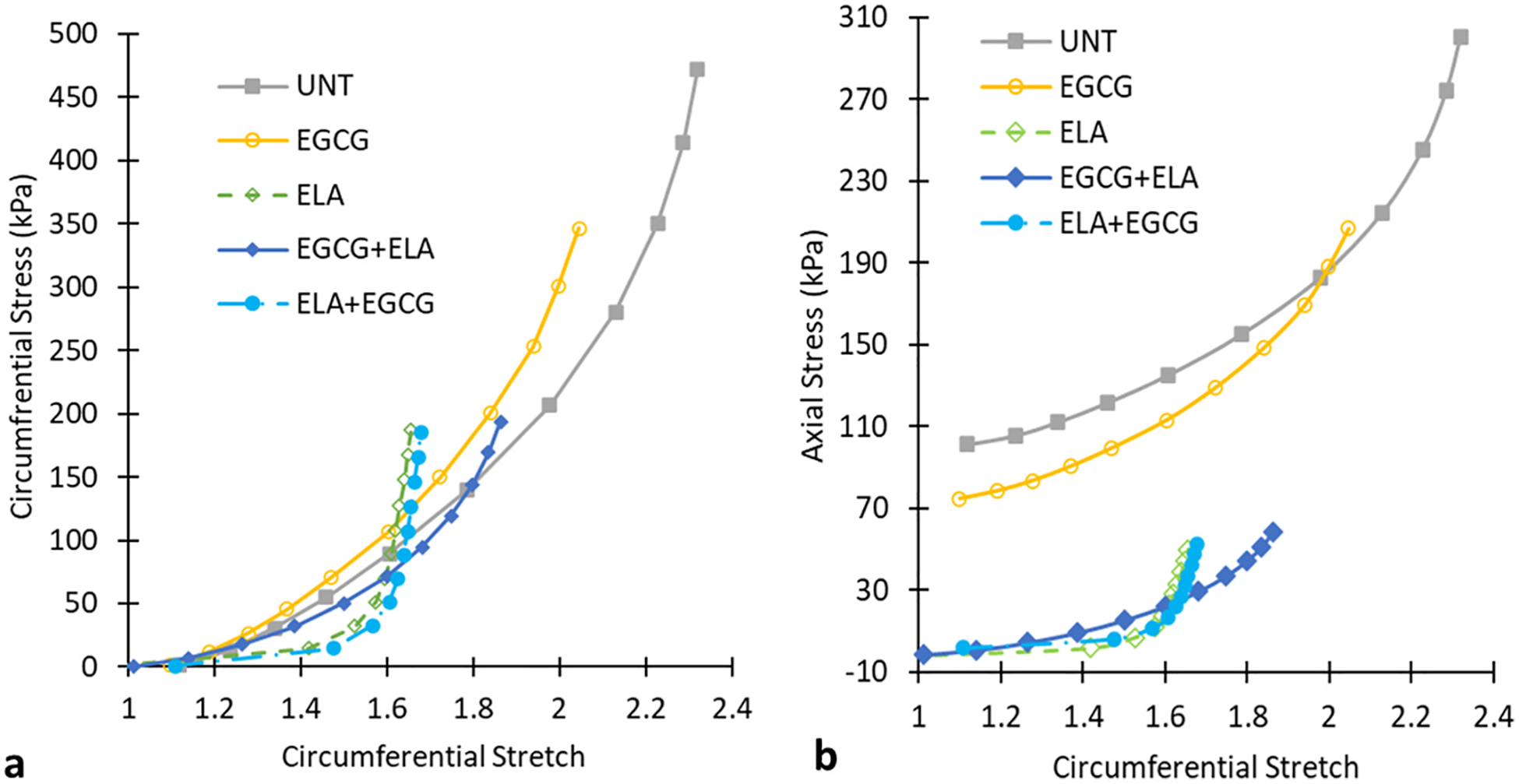
Average circumferential stress-circumferential stretch (a) and axial stress-circumferential stretch (b) of the ATAs for the five groups. Error bars are not shown for clarity as the stress and stretch values vary at each pressure for each ATA. N = 20 for UNT, N = 5/group for all others.

The circumferential and axial stresses were calculated from the mechanical test data and unloaded dimensions. Since the applied stretches and resulting stresses of each ATA depend on the individual unloaded dimensions, the stress-stretch curves were not quantitatively compared. However, qualitatively, Fig. 4a and b show similarities between UNT and EGCG ATAs and between ELA and ELA + EGCG ATAs, with EGCG + ELA ATA behavior in the middle between these two clusters. Comparison of the stress-stretch curves shows that the preventative treatment, EGCG + ELA, is more effective than the restorative treatment, ELA + EGCG, at maintaining the material behavior near UNT ATA, especially in the circumferential direction. The stress-stretch curves indicate that EGCG does not significantly alter the ATA material behavior and partially mitigates changes attributed to elastic fiber degradation when used as a preventative measure.

Like the axial force-pressure behavior (Fig. 2b), the changes in axial stress-circumferential stretch behavior (Fig. 4b) for the ELA treated ATAs are mostly due to the different axial stretch ratios between groups (Fig. 2d). Due to fragility of the ATAs in the axial direction after ELA treatment, they cannot be tested at the same axial stretch ratios as UNT and EGCG ATAs. An increase in axial stretch for the ELA treated ATAs would result in increased circumferential stiffness due to coupling between the circumferential and axial directions. This would further increase the differences observed in the ELA and ELA + EGCG ATAs compared to UNT ATA (Fig. 4a). Another factor to consider is that the ELA, EGCG + ELA, and ELA + EGCG ATAs are all at the same axial stretch ratios after treatment (Fig. 2d), so the axial stresses can be compared. The axial stresses for ELA treated ATAs show that the behavior associated with degraded elastic fibers is partially restored towards UNT behavior with the preventative treatment EGCG + ELA (Fig. 4b).

### 3.4. Circumferential material stiffness

**Fig. 5.**
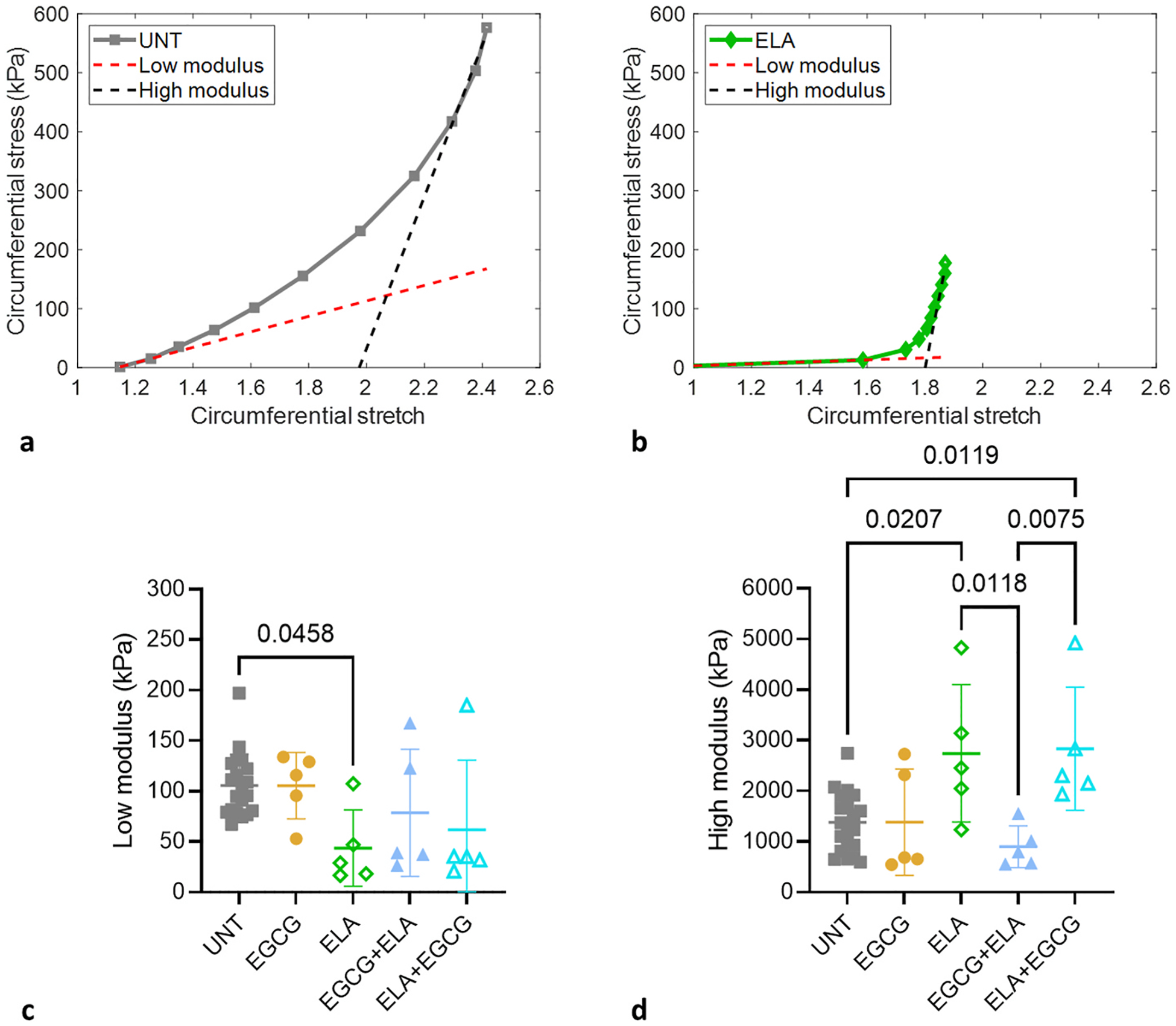
Low and high moduli were determined by fitting linear functions to regions of the circumferential stress-stretch curves. Representative circumferential stress-stretch curves for UNT (a) and ELA (b) ATAs and example linear fits in the low and high pressure ranges. Low (c) and high (d) moduli for each treatment option. Comparisons between groups from one-way ANOVA with Tukey’s posthoc and significant p-values are shown. N = 20 for UNT, N = 5/group for all others.

**Fig. 6.**
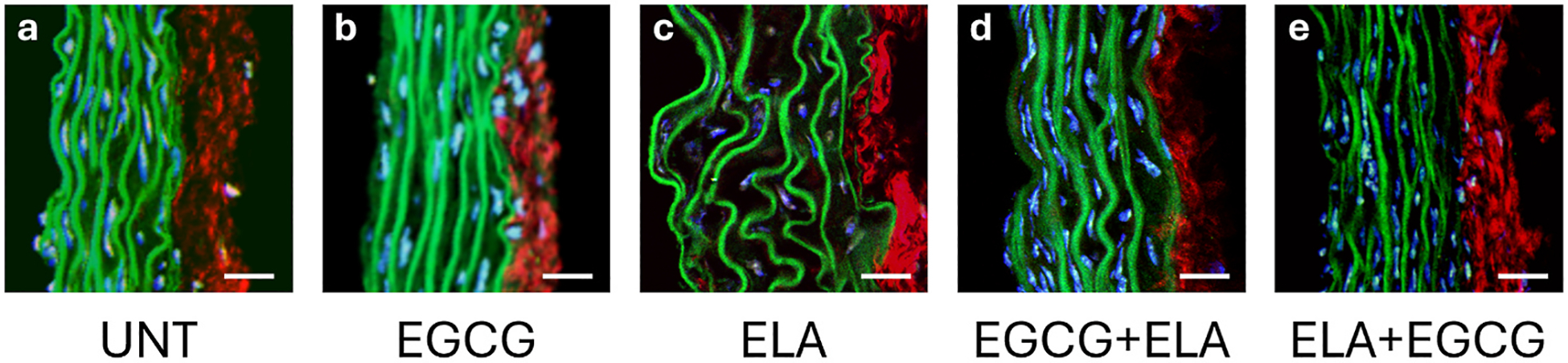
Multiphoton images of ATA cross-sections for UNT (a), EGCG (b), ELA (c), EGCG + ELA (d), and ELA + EGCG (e). Elastin is shown in green, collagen in red, cell nuclei in blue. Images are oriented such that the lumen is at the left. Scale bar = 20 μm. Representative images of N = 2–3 for each group.

The low and high moduli provide information on mechanical contributions typically attributed to elastic and collagen fibers, respectively (Lammers et al., 2008). Representative circumferential stress-stretch curves and fitted lines for the low and high moduli are shown for an UNT ATA in Fig. 5a and for an ELA ATA in Fig. 5b. The low modulus for each treatment is shown in Fig. 6c. The low modulus for ELA ATA is 83 % lower than UNT. The low modulus is not significantly different from UNT for EGCG, EGCG + ELA, or ELA + EGCG ATAs. The high modulus for each treatment is shown in Fig. 6d. The high moduli for ELA and ELA + EGCG ATAs are 66 % and 69 % higher than UNT, respectively. The high moduli for EGCG and EGCG + ELA ATAs are not significantly different from UNT. The results indicate changes to the circumferential material stiffness with ELA treatment that are partially reversed by preventative EGCG + ELA treatment, but not by restorative ELA + EGCG treatment.

### 3.5. Imaging extracellular matrix structure

Multiphoton imaging of ATA cross-sections was performed to qualitatively examine changes in wall structure associated with the different treatments (Fig. 6). The ATAs have layers of elastic fibers (called laminae) alternating with cell nuclei starting at the luminal surface and a collagen layer embedded with cell nuclei at the outer surface. In ELA ATA, the elastic laminae appear thinner and wavier with additional space between each lamina (Fig. 6c). EGCG + ELA ATA (Fig. 6d) appears similar to UNT (Fig. 6a) and EGCG (Fig. 6b). ELA + EGCG (Fig. 6e) appears to be a hybrid of UNT and ELA with thinner, more fragmented laminae than UNT, but less thinning and space between laminae than ELA.

**Fig. 7.**
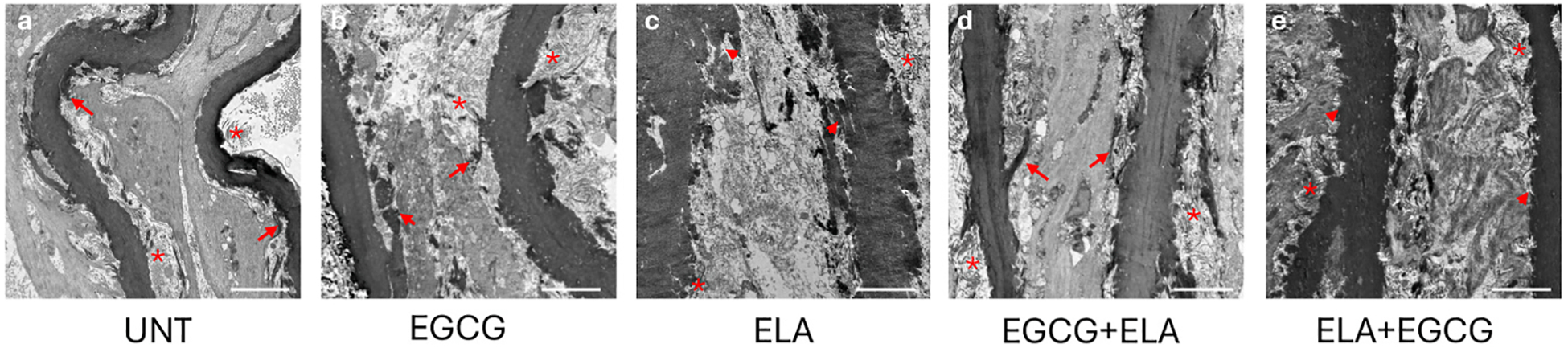
TEM images of ATA cross-sections for UNT (a), EGCG (b), ELA (c), EGCG + ELA (d), and ELA + EGCG (e). Images are oriented such that the lumen is at the left. Elastic laminae connections to adjoining cells are shown with red arrows. Rough, disrupted edges of elastic laminae are shown with red arrowheads. Collagen fibers are shown with red asterisks. Scale bar = 10 μm. Representative images of N = 1–2 for each group.

TEM of ATA cross-sections was performed for a higher resolution qualitative examination of changes in wall structure associated with the different treatments. The changes in elastic fiber structure between groups are subtle even at the resolution of electron microscopy. In the UNT (Fig. 7a), EGCG (Fig. 7b), and EGCG + ELA (Fig. 7c) ATAs the elastic laminae appear as dense, smooth, dark staining layers with clear connections to the adjoining cells. In the ELA (Fig. 7c) and ELA + EGCG (Fig. 7e) ATAs, the elastic laminae edges appear rougher and less dense and the connections between the laminae and the cells are not as obvious, perhaps due to degradation of the outer layer of the elastic laminae by the brief ELA treatment. Collagen fibers can be seen near the elastic laminae in all of the TEM images but are too sparse to determine differences between groups.

## 4. Discussion

Our findings shed light on the potential therapeutic role of EGCG in mitigating cardiovascular disease by preventing the changes in aortic geometry, mechanical behavior, and wall structure associated with elastic fiber fragmentation or degradation. ELA was used to enzymatically degrade mouse ATA elastic fibers in vitro to model cardiovascular diseases such as such as stiffness-induced hypertension (Wagenseil et al., 2012) and aneurysms (Yanagisawa et al., 2020). We found that preventative treatment, EGCG + ELA, where the ATA was treated with EGCG before a brief ELA application, was able to partially prevent mechanical changes observed in ELA ATA and bring mechanical behavior near to UNT ATA. Restorative treatment, ELA + EGCG, was not effective at mitigating changes in mechanical behavior with ELA. Preventative EGCG treatment, and not restorative, also led to ATA geometry and wall structure that was more like UNT ATA than ELA ATA. Although restorative treatments are the most clinically relevant, stiffness-induced hypertension and aneurysms develop over extended periods of time, so partially preventative treatment may be useful. Overall, our results support further investigation of EGCG in preventing geometric, mechanical, and structural changes associated with progressive elastic fiber degradation in the aorta.

### 4.1. Polyphenols and elastic fibers

Polyphenols, like EGCG, increased the rate of tropoelastin self-assembly and elastic fiber production in cell culture (Sinha et al., 2014; Ellis et al., 2022; Chowdhury et al., 2021). The elastogenic potential of EGCG was recently highlighted in a study where prenatal EGCG treatment normalized elastin deposition in an elastin haploinsufficient mouse model and rescued the associated cardiovascular defects (Ellis et al., 2024). Due to its effect on elastogenesis, EGCG has been investigated for its efficacy in preventing aneurysmal dilation associated with elastic fiber degradation. EGCG was administered through drinking water for 2 weeks before elastase/CaCl_2_ induced abdominal aortic aneurysm in rats (Setozaki et al., 2017). EGCG prevented aortic dilation in this model through a combination of increased elastogenesis and decreased inflammation-driven elastic fiber degradation. To our knowledge, direct effects of EGCG on elastic fiber degradation without cellular interaction have not been investigated.

PGG, another polyphenol, increased elastogenesis in cell culture (Sinha et al., 2014; Chowdhury et al., 2021) and also prevented aortic dilation in CaCl_2_ (Isenburg et al., 2007) and elastase (Schack et al., 2020) induced abdominal aortic aneurysm models in rats. Aneurysm prevention by PGG was due partly to stabilization of elastic fibers, as well as multiple other effects similar to EGCG, including increased elastogenesis and decreased inflammation (Patnaik et al., 2019). We showed that PGG stabilized arterial elastic fibers in vitro (without live cells) in preventative (Crandall et al., 2022; Pavey et al., 2021) and restorative (Crandall et al., 2022) treatment protocols. The restorative mechanism for PGG in these in vitro studies is likely PGG binding to elastic fibers and preventing additional degradation associated with multiple loading cycles after ELA treatment (Crandall et al., 2022).

Due to similarities in the polyphenol structures of PGG and EGCG, we hypothesized that EGCG may also be effective at preventing and restoring degraded elastic fibers in vitro without the requirement for live cell interactions. PGG is predicted to bind with high affinity to most vascular proteins (in the wall and circulating) (Simionescu et al., 2023). Similar binding studies of EGCG with multiple vascular proteins have not been performed, but it has been shown to bind to human serum albumin (Bae et al., 2009), membrane receptors (Tachibana et al., 2004), and matrix mettaloproteinases (Sazuka et al., 1997). EGCG has shown promise for cancer prevention (Negri et al., 2018), ameliorating skin photaging (Nagle et al., 2006), and improving cardiovascular and metabolic health (Wolfram, 2007) through these interactions. Our current results show that EGCG prevented aortic mechanical changes associated with elastic fiber degradation in vitro (without live cells), but that it did not work in a restorative fashion like PGG (Crandall et al., 2022). These differences are likely due to subtle structural differences between PGG and EGCG that alter binding to extracellular matrix proteins, like elastic and collagen fibers. Additional work is needed to investigate the role of EGCG in preventing and restoring mechanical changes associated with elastic fiber degradation in vivo or in the presence of live cells. Work in models of abdominal aortic aneurysm (Setozaki et al., 2017) and elastin insufficiency (Ellis et al., 2024) show the promise of EGCG for preventing in vivo elastic fiber degradation and encouraging elastogenesis.

### 4.2. EGCG, elastic fiber degradation, and aortic mechanical behavior

Although EGCG did not restore changes in aortic mechanical behavior after ELA treatment, it did partially prevent changes in aortic mechanical behavior when used before ELA treatment. The mechanism for the preventative EGCG treatment is likely similar to PGG where it binds to vascular wall proteins and prevents access of the ELA. The formation of complexes between arterial elastic laminae and elastin ligands alters metabolic susceptibility to elastase without directly inhibiting elastase catalytic activity (Kagan et al., 1979). Direct binding of EGCG to elastic fibers has not been investigated, as it has for PGG (Isenburg et al., 2006), however EGCG and PGG both bind hydrophobic regions of proteins, such as serum albumin (Kusuda et al., 2006), and may form a layer around the proteins (Dobreva et al., 2011). Both EGCG and PGG can bind collagen and affect collagen structure and crosslinking (Luck et al., 1994; Tedder et al., 2009; Yu et al., 2021), which may also play a role in preventing mechanical changes associated with ELA treatment in this and previous studies (Crandall et al., 2022).

Measures of EGCG + ELA ATA geometry, such as unloaded diameter and unloaded length ratio, mechanical behavior such as diameter-pressure, compliance-pressure, and circumferential stress-stretch relationships, and wall structure, such as qualitative light and electron microscopy, are in between those of UNT/EGCG ATAs and ELA/ELA + EGCG ATAs highlighting the overall efficacy of preventative EGCG treatment in ameliorating geometric, mechanical, and structural changes associated with elastic fiber degradation caused by brief ELA treatment. Preventative EGCG treatment did not block the decrease in low modulus that occurred with ELA treatment and is consistent with the reduction of functional elastic fibers in the aorta (Dwivedi et al., 2024; Kim et al., 2017). However, preventative EGCG treatment did eliminate the increase in high modulus observed with ELA treatment that has been linked to accelerated recruitment of collagen fibers when elastic fibers are degraded (Fonck et al., 2007) or compromised (Dwivedi et al., 2025). The imaging results suggested that subtle changes in aortic wall structure can cause large changes in mechanical behavior and emphasize the importance of mechanical characterization to understand physiologic changes associated with elastic fiber degradation.

When EGCG was delivered in drinking water for 2 weeks before experimental abdominal aortic aneurysm induction in rats, it reduced aortic dilation by 20 %, increased aortic elastin content by over 100 %, and decreased aortic matrix metalloproteinase-9 activity by 70 % compared to untreated controls at 7–28 days after elastase/CaCl_2_ application (Setozaki et al., 2017). Mechanical behavior of EGCG and untreated aortae was not measured. When EGCG was delivered by intraperitoneal injection to pregnant mice, it reduced blood pressure and increased circumferential failure strain of ascending aortic rings in treated *Eln* ^±^ mice compared to untreated controls at one month of age (Ellis et al., 2024). The circumferential Young’s modulus and failure stress of *Eln* ^±^ ascending aortic rings were not different between EGCG and control groups and no other mechanical parameters were measured. These studies support the use of EGCG to induce elastogenesis and reduce elastic fiber fragmentation and degradation in the presence of live cells but provide little information on EGCG effects without cellular interactions or on the resulting aortic mechanical behavior. Our results show that EGCG can partially prevent elastic fiber fragmentation and degradation, even without the presence of live cells, and provide analyses of the resulting circumferential aortic mechanical behavior. Our results also show that EGCG alone does not alter passive mechanical behavior of the mouse ATA.

### 4.3. Limitations

While this study provides valuable insights into the preventative effects of EGCG on elastic fiber degradation and associated mechanical behavior and microstructure of the mouse ATA, there are several limitations that should be acknowledged. We use ELA as a model of elastic fiber degradation, but ELA is not 100 % specific to elastic fibers and likely has additional effects on the ATA wall. The mechanical testing data is limited by assumption of the unloaded dimensions for UNT ATAs, as well as the mismatched axial stretch ratios between treatment groups. We present passive, circumferential mechanical data; additional work is needed to determine the effects of EGCG on active cellular contributions (Wheeler et al., 2015) and biaxial mechanical behavior (Crandall et al., 2022). EGCG dose and treatment time were chosen from preliminary experiments and multiple protocols were not investigated. Our treatment protocol was effective when used in a preventative fashion, but not in a restorative fashion. More experimentation with dose and treatment time is needed to optimize EGCG effects. We used an in vitro model designed to investigate EGCG effects independent of cellular interactions, but additional work with in vivo models would add the beneficial cellular interactions driven by EGCG, as well as be more clinically relevant. In vivo disease models that contain elastic fiber fragmentation include elastase/CaCl_2_ aneurysm induction (Setozaki et al., 2017), as well as aneurysm induction through administration of β-aminoproprionitrile (BAPN) (Weiss et al., 2023), an inhibitor of elastin and collagen crosslinking, along with AngII administration, which induces hypertension, inflammation, and aneurysm in some cases (Bersi et al., 2016; Sawada et al., 2022). Genetic mouse models with fragmented or degraded elastic fibers (Crandall et al., 2023) could be used with EGCG treatment that would eliminate the need for chemical interventions to initiate the disease process.

## 5. Conclusions and future work

We show that EGCG treatment partially prevents geometric, mechanical, and microstructural changes induced in mouse ATA associated with elastic fiber degradation. The in vitro model demonstrates EGCG effects that are independent of cellular interactions. Unlike our previous results with another polyphenol (PGG) (Crandall et al., 2022), EGCG was not effective when used in a restorative treatment protocol. Our structural and mechanical characterization highlight EGCG effects and adds to the literature (Ellis et al., 2024; Setozaki et al., 2017) supporting its use in cardiovascular diseases associated with elastic fiber degradation such as hypertension (Wagenseil et al., 2012) and aneurysms (Yanagisawa et al., 2020).

## Data Availability

Data will be made available on request.
